# Modified Ordered Mesoporous Carbons for Cr(VI) Removal from Wastewater

**DOI:** 10.3390/ma17122881

**Published:** 2024-06-13

**Authors:** Rafał Olchowski, Kinga Morlo, Agnieszka Chałabis-Mazurek, Ryszard Dobrowolski, Katarzyna Tyszczuk-Rotko

**Affiliations:** 1 Department of Pharmacology, Toxicology and Environmental Protection, Faculty of Veterinary Medicine, University of Life Sciences, Akademicka Sq. 12, 20-950 Lublin, Poland; rafal.olchowski@up.lublin.pl (R.O.); agnieszka.mazurek@up.lublin.pl (A.C.-M.); 2 Department of Analytical Chemistry, Faculty of Chemistry, Institute of Chemical Sciences, Maria Curie-Sklodowska University, M. C. Sklodowska Sq. 3, 20-031 Lublin, Poland; kinga.morlo1@gmail.com

**Keywords:** CMK-3, ozonation, hexavalent chromium, redox, galvanic

## Abstract

The pristine CMK-3 carbon was ozonized and then chemically modified by the Zr and Fe compounds. The synthesized carbonaceous materials were characterized with physicochemical methods. The obtained carbons had a high specific surface area (ca. 800 m^2^ g^−1^) and an acidic surface. The Cr(VI) adsorption properties of the oxidized and Zr/Fe-modified carbon were studied. The highest static adsorption capacity towards Cr(VI) ions was evaluated for Zr/Fe-modified carbon (50.1 mg g^−1^) at pH_eq_ = 5.8 after 240 min. The Elovich and Freundlich theoretical models were well fitted to the Cr(VI) adsorption kinetic and isotherm data on the Zr/Fe-modified CMK-3-type carbon. The leading Cr(VI) adsorption mechanism acting on the Zr/Fe-modified carbon was probably based on the redox reactions between Cr(VI) and the carbonaceous surface. Electrostatic attraction and surface complexation processes could also occur during Cr(VI) adsorption in the studied system. The effect of the competitive anions on the concentration level, such as in the galvanic wastewater for Cr(VI) adsorption onto chemically modified carbon, was negligible. The HCl and HNO_3_ media were insufficient for the Zr/Fe-modified carbon regeneration after Cr(VI) adsorption. The Zr/Fe-modified carbon was successfully applied for the efficient (>90%) Cr(VI) removal from the model galvanic wastewater.

## 1. Introduction

Chromium minerals originating from geological processes are subjected to anthropogenic activities, resulting in various chromium species in all components of the ecosystems. Intensive anthropogenic activities such as mining ores, fertilizer applications, tanneries, wastewater irrigation, sewage sludge disposal, the use of pesticides, battery manufacturing, paper industries activities, and vehicular exhaust contribute to the constant increase in environmental pollution with Cr. Generally, Cr is abundant in the Earth’s crust almost in the form of chromium oxide minerals, mainly as chromite (FeCr_2_O_4_), crocoite (PbCrO_4_), and chrome ochre (Cr_2_O_3_). Its wide range of natural sources determines the variety of its forms of occurrence, which affect living organisms in a diversity of ways. Cr(II) has reducing properties, Cr(III) is the most stable oxidation state, and Cr(VI) has strong oxidizing properties. Cr(VI) compounds are considered the most toxic to humans and animals. The primary Cr(VI) forms in an aqueous solution are Cr_2_O_7_^2−^, CrO_4_^2−^, HCrO_4_^−^, and H_2_CrO_4_. On the equilibrium state of various species of chromium in the solution, many parameters have influence: the pH of the solution, the concentration of Cr, the presence of oxidizing and reducing compounds, the redox potential, and finally the kinetics of redox processes. It is worth emphasizing that HCrO_4_^−^ predominates in the pH range of 1 to 6, whereas CrO_4_^2−^ ions are present only if the solution’s pH is higher than 7 [1,2,3,4,5,6,7,8,9].

Considering the toxicity of the Cr(VI) species in the environment, many methods were proposed to remove Cr from wastewater: adsorption, electrochemical treatments, physicochemical processes, biological removal, and membrane filtration. Nowadays, the adsorption method is broadly applied for this purpose due to its low cost, high efficiency, availability, and ease of operation. Various adsorbents with high porosity and that contain on their surface some active centers, which can selectively bind Cr(VI) ions, are described in the literature: algae, bacteria, activated carbon, composites, metal–organic framework, mesoporous silicas, or ordered mesoporous carbons [1].

One of the ordered mesoporous carbons is known as CMK-3. It is obtained with the hard-templating route from the ordered mesoporous silica (SBA-15) and the carbon precursor (such as sucrose). The CMK-3-type carbon contains hexagonally ordered mesopores, which are responsible for the high mass transfer to the surface active centers of this material. Moreover, the CMK-3 carbon has a high specific surface area (ca. 900 m^2^ g^−1^ or higher). It allows for the incorporation of high amounts of the surface active centers, which can selectively bind adsorbates, such as Cr(VI) ions, after properly modifying the pristine carbon. The carbonaceous surface of the CMK-3 carbon can be easily modified by the various compounds (organic functionalities, metal oxides, salts, zero-valent metals, etc.) in different ways (high-temperature treatment, during synthesis approach, on the external surface, or inside the mesopores) [10].

In this paper, the CMK-3-type carbon was, for the first time, oxidized by ozone from the gas phase, and the resulting material was then chemically modified by the Zr and Fe compounds. The synthesized carbons were characterized by the physicochemical methods. Next, the Cr(VI) adsorption optimization on the obtained carbons took place regarding the Cr(VI) aqueous solution pH, the contact time, and the Cr(VI) concentration. The adsorption kinetic and isotherm data were fitted to the selected theoretical models. The effect of the competitive ions (Cl^−^, NO_3_^−^, HCO_3_^−^, SO_4_^2−^, and PO_4_^3−^) on the Cr(VI) adsorption onto the Zr/Fe-modified carbon was studied. Also, the regeneration study concerning Cr-loaded Zr/Fe-modified carbon by HCl and HNO_3_ with different concentrations was performed. The Cr(VI) adsorption mechanism acting on the Zr/Fe-modified carbon was studied in detail. Finally, the synthesized Zr/Fe-modified carbon was applied for Cr(VI) removal from the model galvanic wastewater.

## 2. Materials and Methods

*Reagents used throughout this work* were as follows: ozone generated by the ozone generator (ZY-H102, Grabów, Poland) (ozone flow: 2 L min^−1^, ozone generation efficiency: 500 mg O_3_ h^−1^), nitrogen (99.999%, Air Liquid, Kraków, Poland), ZrOCl_2_·8H_2_O (98%, Warchem, Poland), Fe(NO_3_)_3_·9H_2_O (p. a., Merck, Darmstadt, Germany), NaHCO_3_ (99.7%, Pol-Aura, Zabrze, Poland), NaOH (>95%, Merck, Darmstadt, Germany), KNO_3_ (>99%, Pol-Aura, Zabrze, Poland), NaCl (>99%, Pol-Aura, Zabrze, Poland), KCl (>99%, Pol-Aura, Zabrze, Poland), H_3_PO_4_ (85%, p. a., Pol-Aura, Zabrze, Poland), K_2_SO_4_ (>99%, Pol-Aura, Zabrze, Poland), Zn(NO_3_)_2_ (>99%, Polish Chemical Reagents, Gliwice, Poland), HCl (36%, Suprapur, Merck, Darmstadt, Germany), HNO_3_ (65%, Suprapur, Merck, Darmstadt, Germany), K_2_Cr_2_O_7_ (>99%, Merck, Darmstadt, Germany), double distilled Milli-Q water obtained from Millipore (Merck, Darmstadt, Germany).

*Materials applied during experiments* were as follows: pristine ordered mesoporous carbon (I_CMK-3) synthesized with the hard-templating route according to the procedure described in our previous work [11]; homemade galvanic wastewater (41.5 mg L^−1^ Cr(VI), 4.0 mg L^−1^ Zn(II), 1.0 mg L^−1^ Cl^−^, 1.0 mg L^−1^ SO_4_^2−^, 1.0 mg L^−1^ PO_4_^3−^, pH = 4.41).

**Figure 1:**
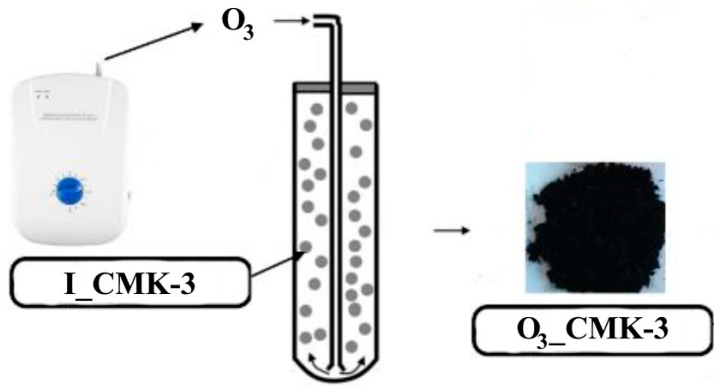
The ozonation of the pristine CMK-3 carbon in the fluidic flow.

*Ozonation procedure of the pristine I_CMK-3 carbon*: An amount of 2 g of the I_CMK-3 carbon were placed into the quartz reactor (Figure 1) and oxidized by the ozone at 25 °C for 24 h in the fluidic flow of the carbon particles. Ozonized carbonaceous material was denoted as O_3__CMK-3.

*Chemical modification of the ozonized carbonaceous material*: An amount of 2 g of the O_3__CMK-3 material was mixed with 500 mL of the distilled water and 2.1 g of the ZrOCl_2_·8H_2_O. The carbonaceous suspension was stirred by the magnetic stirrer (200 rpm min^−1^) for 24 h at 25 °C. Next, the O_3__CMK-3 carbon soaked with the ZrOCl_2_ aqueous solution was filtered and dried in the oven at 120 °C for 24 h. The dried sample was pyrolyzed in the quartz tubular furnace at 800 °C (10 °C min^−1^) for 3 h in the nitrogen flow (1 L min^−1^). Then, 2.7 g of the ozonized CMK-3 carbon modified by the ZrOCl_2_ was mixed with 135.8 mL of an aqueous solution of the Fe(NO_3_)_3_ (10 wt. %). The obtained suspension was stirred at 25 °C for 24 h, the carbonaceous material was filtered, and it was dried at 120 °C for 24 h. Next, it was pyrolyzed in the quartz tubular furnace in nitrogen flow (1 L min^−1^) at 400 °C (10 °C min^−1^) for 2 h. The synthesized material was denoted as OZrFe_CMK-3.

*Physicochemical studies*: The low-temperature (−196 °C) nitrogen adsorption/desorption measurements were conducted with an ASAP 2420 analyzer (Micromeritics, Norcross, GA, USA). The adsorbents were degassed at 120 °C in a vacuum for 14 h before experiments. The desorption branch of the obtained isotherm for each studied sample was used for the estimation of the BJH pore size distribution (PSD) and the following porosity parameters: BET surface area (S_BET_), total pore volume (V_T_), and BJH pore diameter (d_BJH_). The mesopores’ order degree for studied samples was estimated by recording the X-ray diffraction (XRD) patterns with an Empyrean diffractometer (PANalytical, Malvern, UK) equipped with a CuK_α_ radiation source and working with a 0.02° size step and 10 s time step. The morphology studies of the carbonaceous particles and their elemental content were performed with the scanning electron microscope (SEM) Carl Zeiss Ultra Plus (Carl Zeiss, Jena, Germany) equipped with an energy dispersive X-ray (EDX) detector, BrukerAXS (Bruker, Karlsruhe, Germany). This SEM microscope also had secondary electron (SE) and backscattered electron (BSE) detectors. The required conditions (the acceleration voltage: 20 kV, the probe current: 5 nA) were applied during SEM measurements. The Raman spectra were recorded with a dispersive Raman microscope in Via Reflex (Renishaw, Wotton-under-Edge, UK) equipped with an ion-argon laser (514 nm, 20 mW). The CHN elemental analysis was conducted with EA 3000 Elemental Analyzer (Euro Vector, Pavia, Italy). X-ray photoelectron spectroscopy (XPS) measurements were performed with the Multi-Chamber Analytical System (Prevac, Rogów, Poland) equipped with a monochromatic K_α_-Al radiation source (1486.6 eV) (Gammadata Scienta, Uppsala, Sweden) and an X-ray power of 450 W. The carbon C 1s peak at 285 eV was the reference for all binding energies. The zeta potential measurements were performed with Zetasizer Nano ZS (Malvern Instruments, Malvern, UK). For measurements, the proper carbonaceous slurries were prepared (2 mg of carbon and 2 mL of 0.001 mol L^−1^ KCl solution). The pH measurements were performed with the pH meter CP-401 (Elmetron, Zabrze, Poland) equipped with the glass electrode. The acid–base properties of the synthesized carbons were measured by measuring the pH of 0.001 mol L^−1^ KCl (5 mL) that was in contact with the carbonaceous phase (20 mg) for 24 h. The ATR FT-IR spectra of all samples were carried out using the Bruker TENSOR 27 FTIR spectrophotometer (Bruker, Billerica, MA, USA). There were 32 scans accumulated in the range of 600–4000 cm^−1^. The spectrophotometer was equipped with a diamond crystal.

*Cr(VI) adsorption studies*: In each static adsorption experiment, 20 mg of the studied carbonaceous material were immersed in 5 mL of an aqueous Cr(VI) solution with adjusted pH and known Cr(VI) concentration. The obtained carbonaceous slurries were shaken at 170 rpm for 24 h, except for the adsorption kinetics studies. After equilibration, the carbonaceous slurry was centrifuged, and the aqueous solution was separated from the solid phase. The Cr(VI) concentration in the aqueous solution before and after the adsorption process was determined with the flame atomic absorption spectrometer (F AAS) (SpectrAA 880, Varian, Australia) equipped with the Cr hollow cathode lamp (Varian, Australia, lamp current: 7 mA). The operation conditions were as follows: the analytical wavelength: 357.9 nm, the slit width: 0.2 nm, the burner height: 8 mm, the air flow: 13.5 L min^−1^, and the acetylene flow: 3.0 L min^−1^. The Cr(VI) adsorption quantity (A [mg g^−1^]) was calculated as follows:

(1) $$A=\frac{\left(C_{0}-C_{eq}\right)V}{m}$$

where C_0_ is the initial Cr(VI) concentration [mg L^−1^], C_eq_ is the equilibrium Cr(VI) concentration [mg L^−1^], V is the aqueous Cr(VI) solution volume [mL], and m is the adsorbent mass [mg].

The optimization of the Cr(VI) adsorption onto the studied CMK-3-type carbons was conducted by taking into account the solution pH (1–7), contact time (1–1440 min), and Cr(VI) concentration (3–900 mg L^−1^). The pH of an aqueous Cr(VI) solution (58 mg L^−1^) was adjusted by the addition of the proper amounts of the acid (1 mol L^−1^ HNO_3_) or base (1 mol L^−1^ NaOH) aqueous solution. The influence of the contact time for Cr(VI) adsorption onto the synthesized carbons was examined at pH_eq_ = 5.3–5.8 and for 58 mg L^−1^ of the Cr(VI). The Cr(VI) adsorption isotherms on studied carbons were obtained at pH_eq_ = 5.3–5.8, at temperature of 25 °C, and for contact time of 24 h.

*The Cr(VI) adsorption kinetics fitting*: For this purpose, two theoretical models were applied: pseudo-second order (Equation (2)) and Elovich (Equation (3)) [12]. These two models are described by the following equations:

(2) $$\frac{1}{q_{t}}=\frac{1}{q_{eq}}+\frac{1}{q_{eq}^{2}k_{2}t}$$

(3) $$q_{t}=\frac{1}{\beta }\ln\left(\alpha \beta \right)+\frac{1}{\beta }\ln t$$

where q_t_ is the Cr(VI) adsorption at time t [mg g^−1^], q_eq_ is the maximum Cr(VI) adsorption in the studied system [mg g^−1^], t is the time of adsorption [min], k_2_ is the kinetics rate constant for the pseudo-second-order model [g mg^−1^ min^−1^], α is the initial adsorption rate [mg g^−1^ min^−1^], and β is the constant depending on the chemisorption activation energy and the extent of the surface coverage [g mg^−1^].

*The Cr(VI) adsorption isotherm fitting*: For this purpose, the Langmuir (Equation (4)), Freundlich (Equation (5)), and Temkin (Equation (6)) theoretical models were used [12,13,14]. These models are expressed by the following mathematical equations:

(4) $$\frac{1}{q_{eq}}=\frac{1}{q_{m}}+\frac{1}{C_{eq}q_{m}k_{L}}$$

(5) $$\;\ln\left(q_{eq}\right)=\ln\left(k_{F}\right)+n_{F}\ln\left(C_{eq}\right)$$

(6) $$q_{e}=B_{1}\ln C_{eq}+B_{1}\ln k_{T}$$

where q_m_ is the Cr(VI) adsorption capacity [mg g^−1^], C_eq_ is the Cr(VI) equilibrium concentration [mg L^−1^], k_L_ is the Langmuir equilibrium constant [L mg^−1^], k_F_ is the Freundlich equilibrium constant [mg^1−nF^ L^nF^ g^−1^], n_F_ is the Freundlich constant [au], k_T_ is the Temkin equilibrium binding constant [L mg^−1^], and B_1_ is related to the heat of adsorption [J mol^−1^].

*The influence of competitive anions*: This effect was studied for HCO_3_^−^, Cl^−^, SO_4_^2−^, PO_4_^3−^, and NO_3_^−^ anions towards Cr(VI) ions during its adsorption onto the OZrFe_CMK-3 carbon. An amount of 20 mg of the OZrFe_CMK-3 carbonaceous material was mixed with 5 mL of an aqueous solution containing Cr(VI) ions (50 mg L^−1^) and competitive ions in the 0–1000 mol L^−1^ concentration range. The pH of the prepared solutions was adjusted to 5.8. After shaking the slurries for 24 h, initial and equilibrium Cr(VI) concentrations were determined, and the Cr(VI) adsorption was calculated as mentioned above. Finally, the relative adsorption was calculated according to the equation:

(7) $$Adsorption=\frac{A}{A_{max}}100\%$$

where Adsorption is the relative Cr(VI) adsorption onto the OZrFe_CMK-3 carbon [%] and A_max_. is the maximum adsorption of Cr(VI) in the studied adsorption system [mg g^−1^].

*The Cr desorption studies*: Two acids (HCl and HNO_3_) with the concentration range 1–15 mol L^−1^ were tested for Cr removal from the Cr-loaded OZrFe_CMK-3 carbon. Briefly, 5 mg of Cr-loaded (38.3 mg g^−1^) OZrFe_CMK-3 carbon was immersed in 2 mL of the liquid medium for 24 h with shaking of the obtained carbonaceous slurry. After that, the slurry was centrifuged, and the Cr concentration from the upper liquid phase was determined with the F AAS technique, as described above. The desorption degree of the Cr from the Cr-loaded OZrFe_CMK-3 was calculated as follows:

(8) $$Desorption=\frac{C_{Cr}V_{liquid\;medium}}{m_{Cr_{OZrFe}-CMK-3}A_{Cr}}100\%$$

where Desorption is the Cr desorption degree from the studied material [%], C_Cr_ is the Cr concentration in the liquid medium after desorption [mg L^−1^], V_liquid medium_ is the volume of the used liquid medium [mL], m_Cr_OZrFe-CMK-3_ is the mass of Cr-loaded adsorbent [mg], and A_Cr_ is the Cr content in the Cr-loaded OZrFe_CMK-3 carbon [mg g^−1^].

*The Cr(VI) removal from the galvanic wastewater*: An amount of 20 mg OZrFe_CMK-3 carbon was mixed with 5 mL of the galvanic wastewater (Cr(VI): 41.5 mg L^−1^, Zn(II): 4.0 mg L^−1^, Cl^−^: 1 mg L^−1^, SO_4_^2−^: 1 mg L^−1^, PO_4_^3−^: 1 mg L^−1^) with the adjusted pH = 2.5, and it was shaken (150 rpm). Next, the suspension was centrifuged, and in the upper liquid phase, the Cr was determined with the F AAS technique. The Cr(VI) removal efficiency [%] was calculated as follows:

(9) $$Removal\;efficiency=\frac{C_{0\_Cr(VI)}-C_{eq\_Cr(VI)}}{C_{0\_Cr(VI)}}100\%$$

where C_0_Cr(VI)_ is the initial Cr(VI) concentration in the model galvanic wastewater [mg L^−1^] and C_eq_Cr(VI)_ is the equilibrium Cr(VI) concentration in the model galvanic wastewater [mg L^−1^].

## 3. Results and Discussion

### 3.1. Physicochemical Characteristics

**Figure 2:**
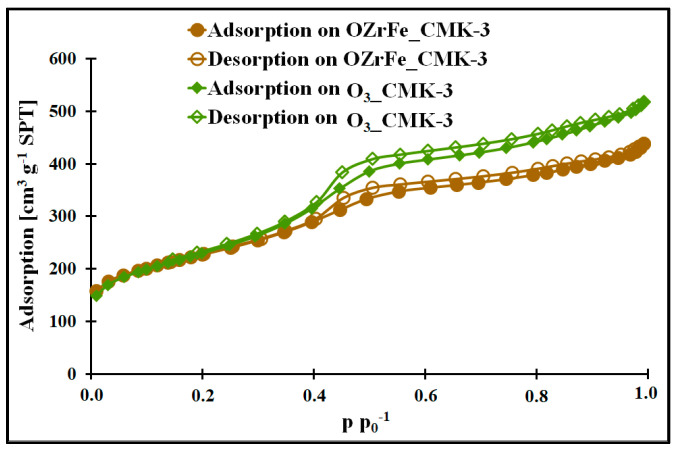
Nitrogen adsorption/desorption isotherms of studied carbons.

In Figure 2 and [11], the nitrogen adsorption/desorption isotherms for all studied mesoporous carbons are depicted. They are classified as Iva type according to the IUPAC [15]. Each isotherm possesses the hysteresis loop of the H1 type at 0.4 p p_0_^−1^. The presence of the hysteresis loop shows the presence of the mesoporous structure with homogenous, narrow, and slit-shaped mesopores. Additionally, the isotherm begins from the high nitrogen adsorption values for all studied carbons, which indicate the strongly developed surface in each case. The mesopores’ presence is confirmed by the BJH pore size distributions of the I_CMK-3 [11], O_3__CMK-3, and OZrFe_CMK-3 carbons (Figure 2), for which the pore diameters are in the range of 3.1–3.8 nm (mesopore region). The BJH pore size distributions for the studied carbons also reveal the microporous structure, which can be related to the carbon rod connectors [16].

**Figure 3:**
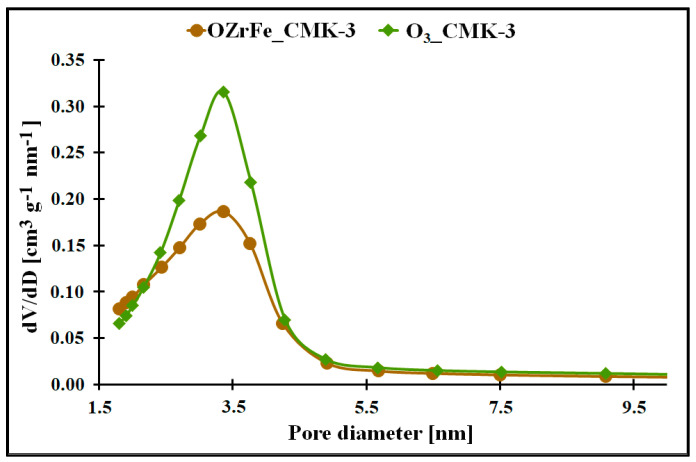
The pore size distribution of the synthesized carbonaceous materials.

The micro-mesoporous structure of the studied mesoporous carbons is confirmed by the porous data obtained from the nitrogen adsorption/desorption isotherms (Table 1, Figure 3). All studied carbons have a high specific surface area (>600 m^2^ g^−1^), which can be related to the presence of micropores in their structure. In contrast, the high total pore volume values (>0.6 cm^3^ g^−1^) are possible due to the mesopores’ presence in the structure of CMK-3, O_3__CMK-3, and OZrFe_CMK-3 carbonaceous materials. The ozone-oxidation of the pristine CMK-3 carbon is responsible for the substantial increase in the S_BET_ value (from 663 m^2^ g^−1^ to 815 m^2^ g^−1^) and slight increase in the V_T_ value (from 0.73 cm^3^ g^−1^ to 0.80 cm^3^ g^−1^) of the resulted material. This oxidation process also changed the mesopore diameter from 3.1 nm to 3.4 nm. It proves that carbon ozonation is an effective process for the microporosity development in the carbonaceous structure, and the formed surface functionalities are probably responsible for the mesopore expansion. The further chemical modification of the O_3__CMK-3 carbon by the Zr and Fe compounds resulted in a small decrease in the S_BET_ (from 815 m^2^ g^−1^ to 798 m^2^ g^−1^), an observable decrease in V_T_ (from 0.80 cm^3^ g^−1^ to 0.68 cm^3^ g^−1^) and no change in the mesopore diameter. These data suggest the introduction of the Zr and Fe compounds inside the mesopores rather than micropores, which decrease the diameter of the mesopores obtained from the mesoporous carbon.

**Figure 4:**
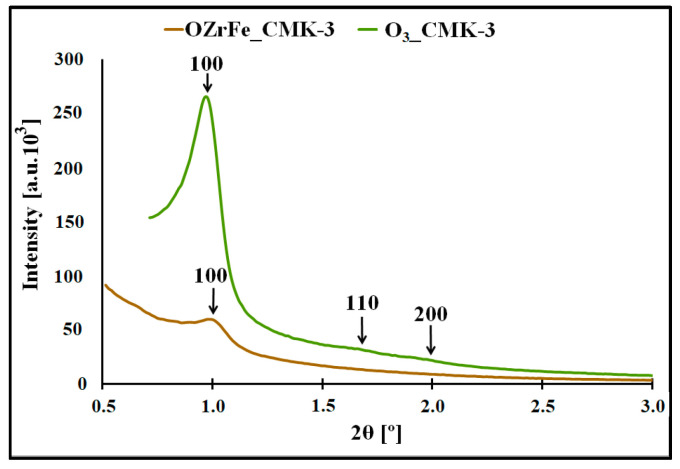
XRD diffractograms registered in the low angle range for studied carbonaceous materials.

In Figure 4, the XRD diffractograms for the O_3__CMK-3 and OZrFe_CMK-3 carbons are presented. In the case of the ozone-oxidized carbon, the three reflections are observed for 2Θ angles 1.0°, 1.7°, and 1.9°, which are related to the hexagonal p6mm symmetry of the mesoporous structure, like those for the pristine I_CMK-3 carbon [11]. Another situation is observed for the OZrFe_CMK-3 carbon, for which only one reflection was recorded for 2Θ angle 1.0°. It means that the ozone-oxidation of the I_CMK-3 carbon does not influence the mesopores’ order degree, in contrast to the Zr and Fe modification of the oxidized carbon material. In the latter case, the order degree of the mesopores severely decreased, which is probably connected with the incorporation of Zr and Fe compounds inside the mesopores of the OZrFe_CMK-3 carbonaceous material.

**Figure 5:**
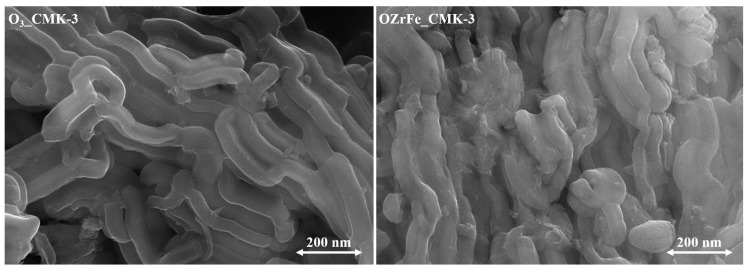
SEM microphotographs of the synthesized carbonaceous materials (magnification 50,000×).

The morphology of CMK-3-type carbon particles was studied with an SEM (Figure 5). The ozonation of the pristine carbonaceous material did not affect the rod-like morphology of carbon nanoparticles [11]. On the contrary, incorporating the Zr and Fe compounds onto the surface of the O_3__CMK-3 carbon resulted in the disorder of the rod-like morphology.

**Figure 6:**
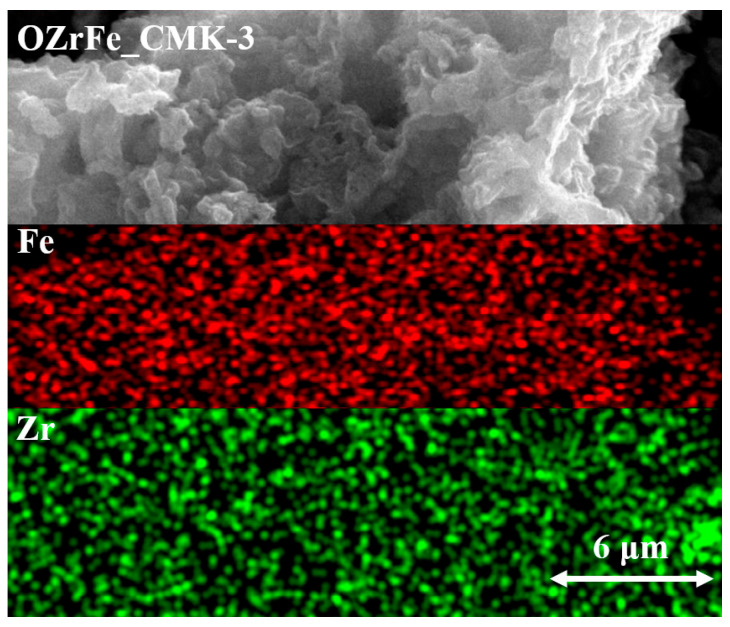
SEM mapping of Zr and Fe for OZrFe_CMK-3 carbon (magnification 10,000×).

Additionally, the results from the SEM mapping of Zr and Fe for OZrFe_CMK-3 have been presented in Figure 6. These two metals are homogenously dispersed on the surface of the OZrFe_CMK-3 carbonaceous material. Also, Zr and Fe are placed in the same places, which can be related to the formation of the mixed Zr-Fe compound on the carbonaceous surface during its chemical modification [17].

**Figure 7:**
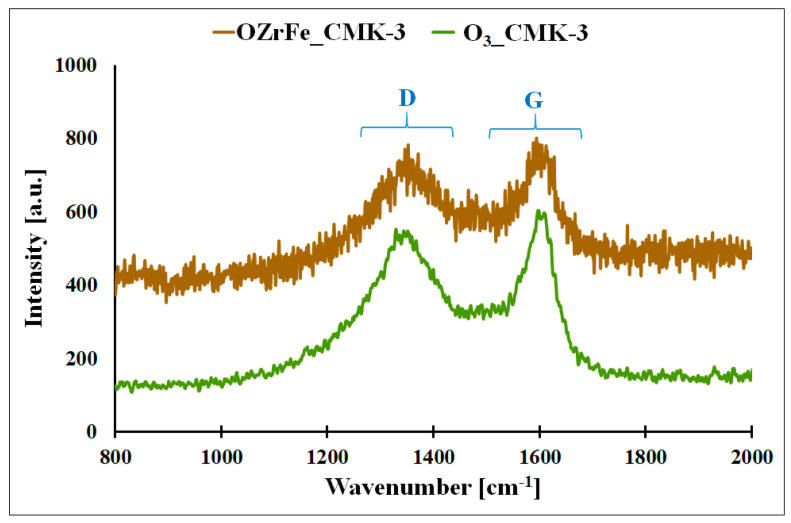
Raman spectra for O_3__CMK-3 and OZrFe_CMK-3 carbonaceous materials (D–D band, G–G band).

In Figure 7, the Raman spectra of the synthesized carbonaceous materials are presented. On these spectra, two bands are observed: D, located at 1317 cm^−1,^, and G, located at 1581 cm^−1^. The D band corresponds to the defects in the carbonaceous material’s graphene domains, and the G band is related to the graphene domains without defects, like those in the pure graphene [11]. The shape of the Raman spectra for O_3__CMK-3 and OZrFe_CMK-3 is similar to the shape of the Raman spectrum for the pristine I_CMK-3 carbon, which is related to the amorphous structure of the carbon rods of these materials [11]. Additionally, the intensity ratio of D and G bands (I_D_ I_G_^−1^) practically did not change after the modification of the pristine and ozonized carbonaceous materials (I_CMK-3: 0.90, O_3__CMK-3: 0.88, OZrFe_CMK-3: 0.89). This means that oxygen and Zr/Fe functionalities were not incorporated into the inner structure of the graphene layers but rather on their edges.

The elemental composition of the synthesized carbons is presented in Table 2. According to the CHN analysis, the carbon content in modified CMK-3-type materials is lower (74.1–76.8 wt. %) than in the case of the pristine carbonaceous material (90.8 wt. %) [11]. It is related to incorporating heteroatoms (O, N) or metals (Fe, Zr) on the carbonaceous surface. The highest H content is observed for the ozonized carbon (0.99 wt. %) and the lowest for the Zr- and Fe-modified carbons (0.72 wt. %). The ozonation of the pristine CMK-3-type material could provide more saturated carbon chains. In turn, the high-temperature modification of the ozonated carbonaceous material with Zr and Fe could favour the formation of unsaturated carbon chains or aromatic carbon rings. Additionally, the incorporation of nitrogen into the carbonaceous structure of O_3__CMK-3 (0.39 wt. %) and OZrFe_CMK-3 (0.70 wt. %) materials could be the result of the nitrogen oxide formation during the ozonation process and the incorporation of this heteroatom from iron(III) nitrate. According to SEM-EDX and XPS analyses, the ozonation of the pristine carbonaceous material resulted in the incorporation of 8.5–10.5 wt. % of O. Moreover, the Zr- and Fe-modification of the O_3__CMK-3 carbon resulted in the introduction of Zr (7.1–13.9 wt. %) and Fe (1.4–5.1 wt. %) onto the carbonaceous surface. The differences in the element content obtained from the CHN, SEM-EDX, and XPS analyses can be related to their inhomogeneous distribution across the depth of the studied materials.

The further investigation of the chemistry of the carbonaceous surface can be carried out by determining the zeta potential and pH values for the carbonaceous suspension in the electrolyte, e.g., 0.001 mol L^−1^ KCl. The ozonation of the CMK-3-type carbon decreased the zeta potential (from −23.3 mV to −33.6 mV) and increased the pH value (from 4.50 to 2.92). Probably during the ozonation process, acidic oxygen functional groups (mainly carboxyl) could be incorporated onto the carbon surface. These functionalities can dissociate in electrolyte solutions. A freed hydrogen cation is responsible for the decrease in the pH of the aqueous solutions, and the carbonaceous surface is negatively charged. In the case of the OZrFe_CMK-3 carbon, the zeta potential and pH values are −17.0 mV and 5.38, respectively. The less negative surface charge of OZrFe_CMK-3 carbon particles than for the ozonized carbonaceous material can result from incorporating Zr and Fe cations onto its surface. Additionally, the high-temperature treatment of the O_3__CMK-3 carbon during Zr- and Fe-modification could be responsible for the effective partial decomposition of the acidic oxygen functionalities; thus, the pH value for this material is higher than for the ozonized carbonaceous sample.

The kind of surface functionalities can be explored by the FT-IR and XPS studies. The FT-IR spectra for studied carbons are presented in Figure S1. For the ozonized carbonaceous material, there are the following bands presented: ν_OH_ (3427 cm^−1^, 2738–2513 cm^−1^), ν_ArH_ (3200–3000 cm^−1^), ν_as,CH3_ (2967–2963 cm^−1^), ν_as,CH2_ (2921–2917 cm^−1^), ν_s,CH3_ (2876 cm^−1^), ν_s,CH2_ (2848–2846 cm^−1^), ν_as, C=C=O_ (2124 cm^−1^), ν_C=O_ (1732–1728 cm^−1^, 1622–1607 cm^−1^), ν_as,_ Ar and δ_s,CH2_ (1447–1440 cm^−1^), δa_s, CH3;C-OH_ (1430 cm^−1^), δ_s,CH3_ (1384 cm^−1^), ν_C-O,C-O-O_ (1166–1046 cm^−1^), and γ_CH3,Ar,ArH_ (876–668 cm^−1^) [18]. The ozonation of the pristine CMK-3 carbonaceous material resulted in the intensity increase of bands corresponding to the OH, CH_2_, CH_3_, C=O, Ar, ArH, and C-O groups. Moreover, the new bands are presented: C=C=O (oxidation of the aromatic carbon rings) and C-O-O (peroxides). The observed changes are related to the content increase in the surface oxygen functionalities in the studied sample (e. g., peroxides, carboxyl groups) and the partial degradation of the graphene domains with the formation of the carbon chains. In the case of the Zr- and Fe-modified CMK-3-type carbon, there are observed the following bands (Figure S1): ν_OH_ (3430 cm^−1^), ν_ArH_ (3200–3000 cm^−1^), ν_as,CH3_ (2971 cm^−1^), ν_as,CH2_ (2927 cm^−1^), ν_s,CH2_ (2849 cm^−1^), ν_C=O_ (1732 cm^−1^, 1627 cm^−1^), ν_as,Ar_ and δ_as, CH3;s,CH2_ (1460 cm^−1^), δ_s,CH3_ (1386 cm^−1^), ν_C-O_ and γ_Zr-OH_ (1052 cm^−1^), γ_Ar,ArH,CH3_ (883–669 cm^−1^), ν_Fe-O_ (582 cm^−1^), and ν_Zr-O_ (442 cm^−1^) [18,19,20,21,22]. Introducing the Zr and Fe compounds onto the ozonized carbon surface resulted in the intensity decrease of bands corresponding to the OH, CH_2_, CH_3_, Ar, ArH, C-O, and C=O groups. Additionally, new bands appeared: Fe-OH, Fe-O, Zr-OH, and Zr-O. It is probably related to the formation of the Zr and Fe oxides/hydroxides onto the carbonaceous surface during the high-temperature modification, the partial degradation of the surface oxygen functionalities, and the vibration damping of the carbon chains and aromatic carbon rings.

In Figure S2, the high-resolution XPS spectra of the core energy levels C 1s (284.8 eV) and O 1s (532.8 eV) for the ozonized carbonaceous material are presented. These two bands were mathematically deconvoluted. In the case of the C 1s band, the following signals were obtained: C=Csp^2^ (284.5 eV), C-Csp^3^ (285.0 eV), C-O/C-N (286.2 eV), C=O (287.0 eV), and O-C=O (288.6 eV) [23]. The deconvolution of the O 1s band resulted in the five signals: O=C (530.9 eV), O=C-O (531.9 eV), and O-C_alif_. (533.0 eV), O-C_arom_. (533.9 eV), and H_2_O, O_2_, _ads_. (535.6 eV) [24,25]. The ozonation of the pristine CMK-3 carbon resulted in the content decrease in C=Csp^2^ (from 91 wt. % to 62 wt. %) and C=O (from 1.6 wt. % to 0.8 wt. %). This process also induced the content increase in C-Csp^3^ (from 3 wt. % to 20 wt. %), C-O/C-N (from 5 wt. % to 8 wt. %), and O=C-O (from 0 wt. % to 3.4 wt. %). The ozone treatment onto the I_CMK-3 surface induced the partial degradation of the aromatic carbon rings and carbonyl groups and the content increase in the aliphatic carbon chains and the hydroxyl and carboxyl groups. The latter is responsible for the acidic surface character of the O_3__CMK-3 carbon.

In Figure S3, the high-resolution XPS spectra of the core energy levels C 1s (284.8 eV), O 1s (530.8 eV), Fe 2p_3/2_ (711.3 eV), and Zr 3d (182.3 eV) for the OZrFe_CMK-3 carbonaceous material are presented. All of these bands were mathematically deconvoluted, and the following signals were obtained: C 1s band: C=Csp^2^ (284.5 eV), C-Csp^3^ (285.1 eV), C-O/C-N (286.3 eV), C=O (287.9 eV), and O=C-O (289.6 eV) [10]; O 1s band: O^2−^, metal oxides (530.1 eV), O=C (530.9 eV), O=C-O (531.9 eV), O-C_alif_. (533.1 eV), O-C_arom_. (534.0 eV), and H_2_O, O_2,ads_. (535.7 eV) [24,25]; Fe 2p_3/2_ band: FeOOH (710.3 eV, 711.2 eV, 712.3 eV, 713.5 eV, 714.5 eV) [26]; and Zr 3d band: ZrO_2_ (182.7 eV and 185.1 eV) [27]. The chemical modification of the O_3__CMK-3 carbon with the Fe and Zr compounds at high temperatures resulted in the content increase in C-Csp^3^ (up to 27 wt. %) and O=C (up to 25.3 wt. %) and the content decrease in O=C-O (up to 2.6 wt. %) and O-C (up to 11.5–17.4 wt. %). Additionally, the FeOOH and ZrO_2_ particles were introduced onto the carbonaceous surface during modification. The Fe and Zr surface active centers can significantly enhance the adsorption properties of the OZrFe_CMK-3 carbon towards Cr(VI) ions.

### 3.2. Cr(VI) Adsorption Optimization

**Figure 8:**
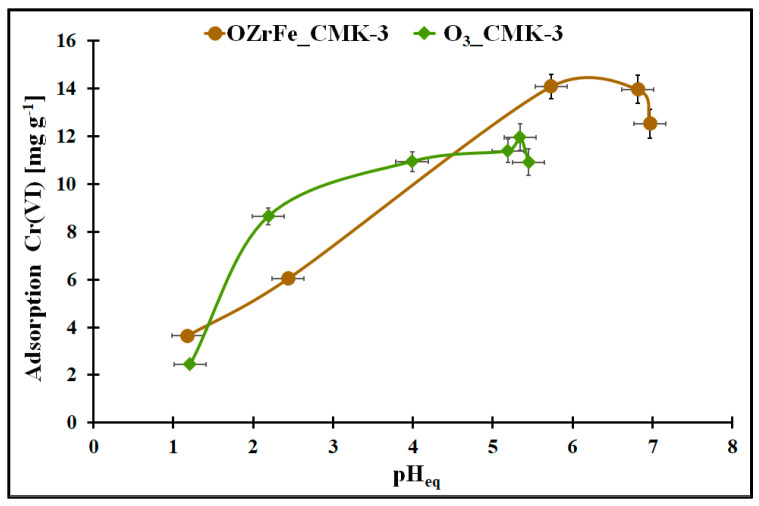
The Cr(VI) adsorption in the function of the pH_eq_ for the studied carbonaceous materials (m = 20 mg, V = 5 mL, t = 24 h, C_0Cr(VI)_ = 58 mg L^−1^).

The changes in the Cr(VI) adsorption onto the studied carbons in the function of the pH_eq_ are illustrated in Figure 8. In the case of the ozonized carbon, the pH_eq_ change from 1.0 to 5.3 is related to the Cr(VI) adsorption increase from 2.5 mg g^−1^ to the maximum value in the studied conditions (ca. 12 mg g^−1^). The further increase in the Cr(VI) aqueous solution pH in this adsorption system induced a slight decrease in the Cr(VI) adsorption (up to 11 mg g^−1^). For OZrFe_CMK-3, the Cr(VI) adsorption increase (from 3.8 mg g^−1^ to 14 mg g^−1^) is observed in the pH_eq_ range from 1.0 to 5.8 and then is constant up to 6.7. Above pH_eq_ = 6.7, the Cr(VI) adsorption decreased to 12.5 mg g^−1^ in the studied adsorption system. Thus, the optimal pH of the aqueous Cr(VI) solution is 5.3 for the O_3__CMK-3 carbon and 5.8–6.7 for the OZrFe_CMK-3 carbon.

In Figure S4, the pH_0_ in the function of the pH_eq_ during the Cr(VI) adsorption for both studied adsorption systems was presented. In the pH_0_ range 1.0–4.0, pH_eq_ > pH_0_ for the O_3__CMK-3 carbon was observed. The pH change during Cr(VI) adsorption in the considered pH_0_ range can be related to the adsorption of the protons from the aqueous Cr(VI) solution onto the carbonaceous surface. Thus, the ozonized carbonaceous surface is positively charged for pH < 2.92. Additionally, the Cr(VI) is present in an aqueous solution at pH < 2, mainly as H_2_CrO_4_, and at 2 < pH < 6.5, mainly as HCrO_4_^−^ anions. HCrO_4_^−^ anions are probably electrostatically attracted to the carbonaceous surface, in contrast to the non-charged Cr(VI) chemical form [28]. The protons participate in the reaction with the HCrO_4_^−^ anions according to the following chemical reaction [29]:

HCrO_4_^−^ + 7H^+^ + 3e^−^ → Cr^3+^ + 4H_2_O (10)

Initially, the formed Cr^3+^ cations are probably electrostatically repulsed from the positively charged carbonaceous surface, which is related to the limited adsorption of this element. As the pH_0_ increases, the carbonaceous surface is less positively charged, and the chromium can be more efficiently adsorbed. These cations can also be trapped by the surface groups due to the formation of the surface complexes with hydroxyl, carbonyl, or carboxyl functional groups on the O_3__CMK-3 surface. For pH_0_ > 4, the buffer effect is observed (e.g., the constant pH_eq_ value (5.3) despite the pH_0_ increase). This is also related to practically no change in Cr(VI) adsorption onto the studied carbon. In these conditions, the surface of the ozonized carbonaceous material is negatively charged, and in an aqueous solution, Cr(VI) is present in anionic forms. The electrostatic repulsion can occur between the carbonaceous surface and Cr(VI) anions.

In the case of the OZrFe_CMK-3 carbon, the pH_0_ and pH_eq_ changes during Cr(VI) adsorption onto its surface are similar, but the adsorption process is more efficient. It can be related to the presence of the metal cations on the surface of the OZrFe_CMK-3 carbon, which can shift the point of zero charge (5.38), and they can be the surface active sites for Cr(VI) anions to bind them by the electrostatic attraction and the surface complexation [30].

**Figure 9:**
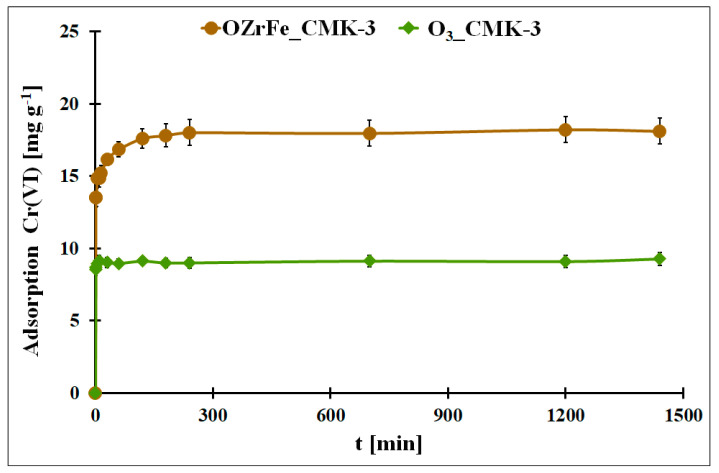
The Cr(VI) adsorption in the function of the contact time for the studied carbonaceous materials (m = 20 mg, V = 5 mL, pH_eq_O3_CMK-3_ = 5.3, pH_eq_OZrFe_CMK-3_ = 5.8, C_0Cr(VI)_O3_CMK-3_ = 58 mg L^−1^, C_0Cr(VI)_OZrFe_CMK-3_ = 74 mg L^−1^).

In Figure 9, the adsorption kinetics for Cr(VI) onto O_3__CMK-3 and OZrFe_CMK-3 carbonaceous materials are presented. In both cases, the adsorption equilibrium is achieved quickly: after 10 min (O_3__CMK-3) and after 240 min (OZrFe_CMK-3). The fast equilibration can be due to the ordered mesopores in the structure of both studied carbonaceous materials. Moreover, the Cr(VI) adsorption process is one-step in the case of the O_3__CMK-3 carbon and two-step for the OZrFe_CMK-3 carbon. The two-step Cr(VI) adsorption for the OZrFe_CMK-3 carbon can be related mainly to the chemical reaction between the Cr(VI) ions and the carbonaceous surface, which can be the driving force for this process.

The experimental Cr(VI) adsorption kinetic data were fitted to two theoretical models: pseudo-second order and Elovich (Table 3) [12]. In the case of the ozonized CMK-3-type carbon, the pseudo-second-order model better described the adsorption kinetic process (R^2^ = 0.5228). This means that this process was driven by a chemical reaction on the carbonaceous surface. The very high value of the α parameter from the Elovich equation is evidence of the high initial adsorption rate, probably induced by the ordered mesoporous structure of the O_3__CMK-3 carbon. The high β value is also related to the high chemisorption activation energy during Cr(VI) adsorption onto the ozonized carbon.

On the contrary, the Cr(VI) adsorption kinetics onto the OZrFe_CMK-3 carbon is better described by the Elovich model (R^2^ = 0.9165). This means that this process is two-step and controlled by chemisorption. Moreover, the Elovich constant values are significantly lower in this case than for the adsorption system with the O_3__CMK-3 carbon. It can be related to the second lower step during Cr(VI) adsorption onto the surface of OZrFe_CMK-3 and a stronger interaction between the Cr(VI) ions and the carbonaceous surface than for the ozonized carbon.

**Figure 10:**
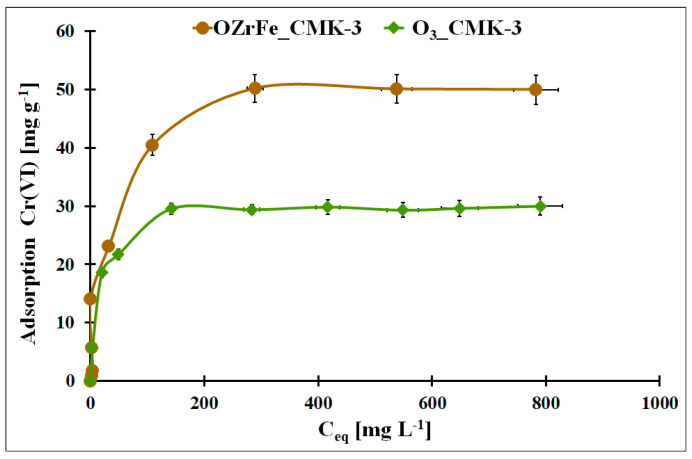
The Cr(VI) adsorption isotherms for studied carbonaceous materials (m = 20 mg, V = 5 mL, pH_eq_O3_CMK-3_ = 5.3, pH_eq_OZrFe_CMK-3_ = 5.8, t_eq_ = 24 h, T = 25 ± 4 °C).

In Figure 10, the Cr(VI) adsorption isotherms for the O_3__CMK-3 and OZrFe_CMK-3 carbons were presented. The highest static adsorption capacity towards Cr(VI) was obtained for the OZrFe_CMK-3 carbonaceous material (50 mg g^−1^). The Cr(VI) adsorption isotherm is paraxially up to 15 mg g^−1^, unlike the ozonized carbon. It can be related to the stronger shift of the Cr(VI) adsorption equilibrium towards the OZrFe_CMK-3 surface than for the O_3__CMK-3 surface and the more efficient Cr(VI) removal from the wastewater. Thus, the OZrFe_CMK-3 carbon was selected for Cr(VI) removal from the galvanic wastewater.

In Table 4, the fitting results of the experimental Cr(VI) adsorption isotherms with the theoretical Langmuir, Freundlich, and Temkin models were presented [12,13,14]. For the ozonized CMK-3-type carbon, the Langmuir model was better fitted to the Cr(VI) adsorption data (R^2^ = 0.9977). This is evidenced by the monolayer and chemical adsorption of the Cr(VI) ions onto this mesoporous carbon. In the case of the OZrFe_CMK-3 carbon, the Temkin model was better for describing the Cr(VI) adsorption isotherm (R^2^ = 0.9606). Additionally, the higher n_F_ factor for this material was estimated (0.61) than for the ozonized carbon (0.26), which means that the latter material possessed a more energetically heterogeneous surface than the first one. Moreover, the Cr(VI) adsorption onto the OZrFe_CMK-3 carbonaceous surface was a multi-layer process. Presented here, the adsorbents were compared with those from the literature in Table S1.

### 3.3. Effect of Competitive Ions

**Figure 11:**
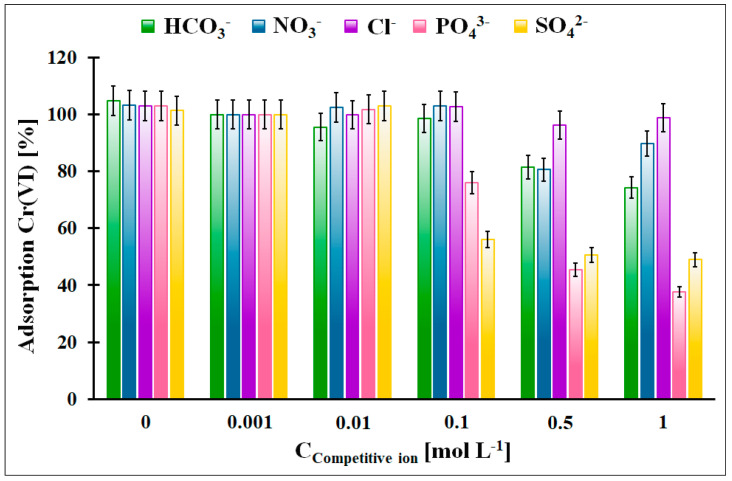
The influence of the selected competitive anions for the relative Cr(VI) adsorption onto the OZrFe_CMK-3 carbon (m = 20 mg, V = 5 mL, pH_eq_ = 5.8, t_eq_ = 24 h, T = 25 ± 4 °C, C_0Cr(VI)_ = 50 mg L^−1^, A_100%_ = 14.11 mg g^−1^).

The influence of the competitive anions, which can be present in the galvanic wastewater, on the Cr(VI) adsorption onto the OZrFe_CMK-3 carbon was illustrated in Figure 11. According to the obtained data, the Cl^−^, NO_3_^−^, and HCO_3_^−^ anions did not influence the Cr(VI) adsorption in the studied adsorption system. The highest impact was observed in the case of the SO_4_^2−^ anions (the 50% decrease in the Cr(VI) adsorption from 100 mmol L^−1^) and PO_4_^3−^ anions (the 60% decrease in the Cr(VI) adsorption from 500 mmol L^−1^). The influence of the SO_4_^2−^ can be explained by the complexation of the Zr(IV) ions on the carbonaceous surface and the strong impact of PO_4_^3−^, which can probably be related to the surface precipitation of the FePO_4_. The formed layer of the adsorbed anions onto the carbonaceous surface can electrostatically repulse the Cr(VI) ions, which resulted in the lower Cr(VI) adsorption onto the studied material.

### 3.4. Adsorbent Regeneration Studies

**Figure 12:**
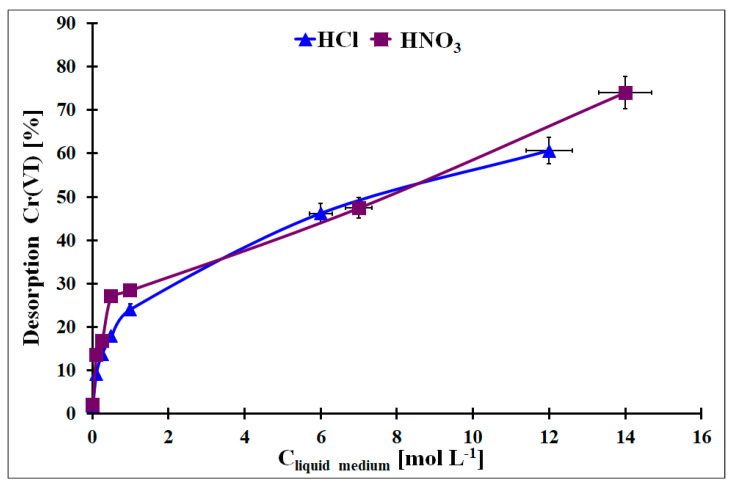
The desorption of the adsorbed Cr from the OZrFe_CMK-3 surface by the HCl and HNO_3_ with various concentrations (m_Cr-loaded carbon_ = 5 mg, V_liquid medium_ = 2 mL, t_des_. = 24 h, A = 38.3 mg g^−1^).

From a practical point of view, the regeneration of the used adsorbent is one of the important factors that classify it for practical application. It enables lower costs of the adsorbent usage and simplifies the removal procedure. In Figure 12, the Cr desorption results from the Cr-loaded OZrFe_CMK-3 carbon with HCl or HNO_3_ with various concentrations were presented. As can be seen, a higher acid concentration induced higher Cr removal. However the Cr desorption from the studied carbon by these acids was not quantitative (max. 80% desorption for the concentrated HNO_3_). This problem with Cr desorption can be related to the Cr presence in micropores, and liquid medium molecules can have limited access to the adsorbed Cr species. Other liquid media should be studied for an efficient Cr desorption from this material to regenerate the OZrFe_CMK-3 carbon.

### 3.5. Cr(VI) Adsorption Mechanism

Further studies of the Cr(VI) adsorption mechanism acting on the OZrFe_CMK-3 carbon were conducted. In Table S1, the XPS elemental composition of the Cr-loaded OZrFe_CMK-3 (50 mg g^−1^ of Cr) was presented. Comparing these data with those for the OZrFe_CMK-3 carbon before Cr(VI) adsorption, it can be seen that the C and Zr content decreased (about 7 wt. % in both cases), O content increased (up to 17.0 wt. %), and N (3.1 wt. %) and Cr (2.5 wt. %) appeared. The appearance of the N on the surface of the studied carbon after Cr(VI) adsorption can be related to the nitric acid present in Cr(VI) aqueous solutions. The changes in the O and Cr content on the OZrFe_CMK-3 surface can result from the Cr(VI) ion adsorption and the carbonaceous surface oxidation processes. The difference in the Zr content before and after Cr(VI) adsorption can be related to the inhomogeneous distribution of this element on the carbonaceous surface. Based on the high-resolution XPS C 1s, O 1s, and Cr 2p_3/2_ data of the OZrFe_CMK-3 carbon after Cr(VI) adsorption (Figure S5), it can be observed that on the studied carbonaceous surface, the Cr(III) is present, and the surface is partly oxidized (an appearance of the carbonates and higher content of carbonyl and carboxyl groups). It suggested that the leading Cr(VI) adsorption mechanism acting on the OZrFe_CMK-3 carbon can be the carbonaceous surface oxidation and simultaneous reduction in the Cr(VI) to the Cr(III) ions. Moreover, electrostatic attraction and surface complexation can also take place.

### 3.6. Cr(VI) Removal from the Model Galvanic Wastewater

The OZrFe_CMK-3 carbonaceous material was applied to remove Cr(VI) from the simulated galvanic wastewater. The galvanic wastewater can contain not only Cr(VI) (up to some hundreds of mg L^−1^) but also other heavy metal ions (such as Zn(II)). These toxic ions can go into environmental water and induce toxic effects on the flora and fauna. Thus, directly removing the Cr(VI) from the galvanic wastewater is a crucial task [31]. As can be seen in Table 5, the 91.2 wt. % of Cr(VI) present in the model galvanic wastewater was removed from this medium by the OZrFe_CMK-3 carbonaceous material. Moreover, according to the previously presented XPS data, the Cr(VI) ions were reduced on the carbonaceous surface to the less harmful Cr(III) ions. The lack of the quantitative removal of Cr(VI) ions from the studied wastewater could be caused by the limited amount of the surface active sites onto OZrFe_CMK-3 and the competitive ions in the galvanic wastewater.

## 4. Conclusions

The novel ozonized and Fe/Zr-modified CMK-3-type carbonaceous materials were synthesized and characterized by various physicochemical methods. The obtained materials possessed a high specific surface area (ca. 800 m^2^ g^−1^). The ozonation process of the pristine CMK-3 carbon is not influenced by the mesopores’ order degree and the morphology of the carbonaceous particles, in contrast to the Zr/Fe-modified carbon. The carbonaceous surface of the synthesized materials was acidic and contained some oxygen or oxygen and Zr/Fe functionalities, constituting the Cr(VI) adsorption active centers.

The best performance for Cr(VI) adsorption from an aqueous solution was evaluated for the Zr/Fe-modified CMK-3-type carbon, with a high static adsorption capacity (50.1 mg g^−1^) at pH_eq_ = 5.8 after 240 min. The Cr(VI) adsorption kinetics onto this carbon material was two-step and well described by the Elovich equation. The Cr(VI) adsorption isotherm on the studied carbon was best fit with the Freundlich model, regarding the multi-layered chemisorption onto the energetically inhomogeneous surface.

The leading Cr(VI) adsorption mechanism acting on the Zr/Fe-modified carbon was probably related to the reduction in the Cr(VI) to the Cr(III) and the simultaneous oxidation of the carbonaceous surface. Also, the electrostatic attraction and the surface complexation of the Cr(VI) and Cr(III) ions could occur during adsorption.

The competitive anions in the galvanic wastewater had practically no effect on the Cr(VI) adsorption onto the studied material.

The HCl and HNO_3_ media do not allow for the quantitative desorption of the Cr from the Cr-loaded adsorbent; thus, other media should be searched for an efficient regeneration of the Zr/Fe-modified carbon after Cr(VI) adsorption.

The Zr/Fe-modified carbon was successfully applied for efficient Cr(VI) removal from the model galvanic wastewater (>90%).

## Figures and Tables

**Table 1:** Porosity parameters (S_BET_—the specific surface area [m^2^ g^−1^], V_T_—the total pore volume [cm^3^ g^−1^], d_BJH_—the pore diameter estimated by BJH method) for studied carbons calculated from the nitrogen adsorption/desorption isotherm data.

Sample	S_BET_ [m^2^ g^−1^]	V_T_ [cm^3^ g^−1^]	d_BJH_ [nm]
I_CMK-3	663 * ± 7 ^&^	0.73 * ± 0.01 ^&^	3.1 * ± 0.1 ^&^
O_3__CMK-3	815 * ± 15 ^&^	0.80 * ± 0.01 ^&^	3.4 * ± 0.1 ^&^
OZrFe_CMK-3	798 * ± 11 ^&^	0.68 * ± 0.01 ^&^	3.4 * ± 0.1 ^&^

*—the mean value from 3 independent measurements, ^&^—the standard deviation from 3 independents measurements.

**Table 2:** The elemental composition of the studied carbonaceous materials obtained from the XPS, CHN, and SEM-EDX analyses.

	Material Symbol	
XPS	O_3__CMK-3	OZrFe_CMK-3
C [wt. %]	88.8 * ± 1.3 ^#^	74.7 * ± 0.4 ^#^
O [wt. %]	10.5 * ± 0.1 ^#^	9.2 * ± 0.1 ^#^
*Fe* [wt. %]	-	1.40 * ± 0.03 ^#^
Zr [wt. %]	-	13.9 * ± 0.7 ^#^
**CHN**	**O_3__CMK-3**	**OZrFe_CMK-3**
C [wt. %]	76.8 * ± 1.6 ^#^	74.1 * ± 1.5 ^#^
H [wt. %]	0.99 * ± 0.03 ^#^	0.72 * ± 0.01 ^#^
N [wt. %]	0.39 * ± 0.04 ^#^	0.70 * ± 0.01 ^#^
**SEM-EDX**	**O_3__CMK-3**	**OZrFe_CMK-3**
C [wt. %]	90.9 * ± 1.7 ^#^	75.1 * ± 0.7 ^#^
O [wt. %]	8.5 * ± 0.1 ^#^	10.6 * ± 0.5 ^#^
N [wt. %]	-	1.14 * ± 0.05 ^#^
Fe [wt. %]	-	5.1 * ± 0.2 ^#^
Zr [wt. %]	-	7.1 * ± 0.4 ^#^

*—the mean value from 3 independent measurements, ^#^—the standard deviation from 3 independents measurements, “-”—no data.

**Table 3:** The results of fitting Cr(VI) adsorption kinetic experimental data with theoretical models pseudo-second order and Elovich for the studied carbons.

Pseudo-Second Order			
Material Symbol	R^2^	q_eq_ [mg g^−1^]	k_2_ [g mg^−1^ min^−1^]
O_3__CMK-3	0.5228	9.05 * ± 0.37 ^#^	1.80 * ± 0.04 ^#^
OZrFe_CMK-3	0.6389	16.75 * ± 0.71 ^#^	0.19 * ± 0.01 ^#^
**Elovich**			
**Material Symbol**	**R^2^**	**α** **[mg g^−1^ min^−1^]**	**β** **[g mg^−1^]**
O_3__CMK-3	0.4685	4.4 10^59^ *^$^	16.1 * ± 0.58 ^#^
OZrFe_CMK-3	0.9165	2.5 10^7^ *^$^	1.29 * ± 0.03 ^#^

*—the mean value from 3 independent measurements, ^#^—the standard deviation from 3 independents measurements, ^$^—the standard deviation close to zero.

**Table 4:** The results of fitting Cr(VI) adsorption isotherm experimental data with theoretical models Langmuir, Freundlich, and Temkin for the studied carbons.

Langmuir				
Material Symbol	R^2^	q_m,t_. [mg g^−1^]	q_m,e_. [mg g^−1^]	k_L_ [L mg^−1^]
O_3__CMK-3	0.9977	30.3 * ± 0.8 ^#^	29.9 * ± 0.3 ^#^	0.07 *^$^
OZrFe_CMK-3	0.5352	27.2 * ± 1.2 ^#^	50.1 * ± 0.4 ^#^	0.03 *^$^
**Freundlich**				
**Material Symbol**	**R^2^**	**n_F_** **[au]**		**k_F_** **[mg^1−nF^ L^nF^ g^−1^]**
O_3__CMK-3	0.8046	0.26 *^$^		6.18 * ± 0.12 ^#^
OZrFe_CMK-3	0.8218	0.61 *^$^		1.48 * ± 0.05 ^#^
**Temkin**				
**Material Symbol**	**R^2^**	**B** **[J mol^−1^]**		**k_T_** **[L mg^−1^]**
O_3__CMK-3	0.8917	4.20 * ± 0.12 ^#^		2.57 * ± 0.11 ^#^
OZrFe_CMK-3	0.9606	8.84 * ± 0.36 ^#^		0.58 * ± 0.01 ^#^

*—the mean value from 3 independent measurements, ^#^—the standard deviation from 3 independents measurements, ^$^—the standard deviation close to zero.

**Table 5:** The results of the Cr(VI) removal from the model galvanic wastewater by the OZrFe_CMK-3 carbonaceous material (m = 20 mg, V = 5 mL, pH_eq_ = 2.5, t_eq_ = 24 h, T = 25 ± 4 °C).

Sample	C_0_Cr(VI)_ [mg L^−1^]	C_eq_Cr(VI)_ [mg L^−1^]	Cr(VI) Removal Efficiency [%]
model galvanic wastewater	41.5 ^#^ ± 1.0 *	3.65 ^#^ ± 0.07 *	91.2 ^$^

*—the mean value from 3 independent measurements, ^#^—the standard deviation from 3 independents measurements, ^$^—the standard deviation close to zero.

## Data Availability

The original contributions presented in the study are included in the article/Supplementary Materials, further inquiries can be directed to the corresponding authors.

## Supplementary Materials

The following supporting information can be downloaded at: https://www.mdpi.com/article/10.3390/ma17122881/s1, Figure S1: The FT-IR spectra of the I_CMK-3, O_3__CMK-3 and OZrFe_CMK-3; Figure S2: The high-resolution XPS spectra of the core energy levels C 1s and O 1s for O_3__CMK-3 carbon; Figure S3: The high-resolution XPS spectra of the core energy levels C 1s, O 1s, Fe 2p_3/2_ and Zr 3d for OZrFe_CMK-3 carbon; Figure S4: The pH_0_ in the function of pH_eq_ for studied carbonaceous materials during Cr(VI) adsorption (m = 20 mg, V = 5 mL, t = 24 h, C_0Cr(VI)_ = 58 mg L^−1^); Figure S5: The Cr 2p_3/2_ energy core level for Cr-loaded OZrFe_CMK-3 carbon (the Cr content: 50 mg g^−1^; A: 576.5 eV, B: 577.5 eV, C: 578.3 eV, D: 579.2 eV, E: 580.0 eV; this signal is related to the Cr(III)); Table S1: The comparison of Cr(VI) adsorption performance between data obtained from the literature and our studies. References [32,33,34] are cited in the Supplementary Materials.
