# Supplementary material for: Modified Ordered Mesoporous Carbons for Cr(VI) Removal from Wastewater
Source: Materials (Basel). 2024 Jun 13;17(12):2881. doi: 10.3390/ma17122881 (PMC11204451; doi:10.3390/ma17122881)
Supplement: Supplementary file 1 [file materials-17-02881-s001.zip › materials-3039740-supplementary.pdf]

materials

MDPI

Supplementary Materials

Modified Ordered Mesoporous Carbons for Cr(VI) Removal from Wastewater

Rafał Olchowski [1], Kinga Morlo [2], Agnieszka Chałabis-Mazurek [1], Ryszard Dobrowolski [2,*] and Katarzyna Tyszczuk-Rotko [2,*]

[1] Department of Pharmacology, Toxicology and Environmental Protection, Faculty of Veterinary Medicine, University of Life Sciences, Akademicka Sq. 12, 20-950 Lublin, Poland
[2] Department of Analytical Chemistry, , Faculty of Chemistry, Institute of Chemical Sciences, Maria Curie-Sklodowska University, M. C. Sklodowska Sq. 3, 20-031 Lublin, Poland
* Correspondence: rdobrow@poczta.umcs.lublin.pl (R.D.); katarzyna.tyszczuk-rotko@mail.umcs.pl (K.T.-R.)

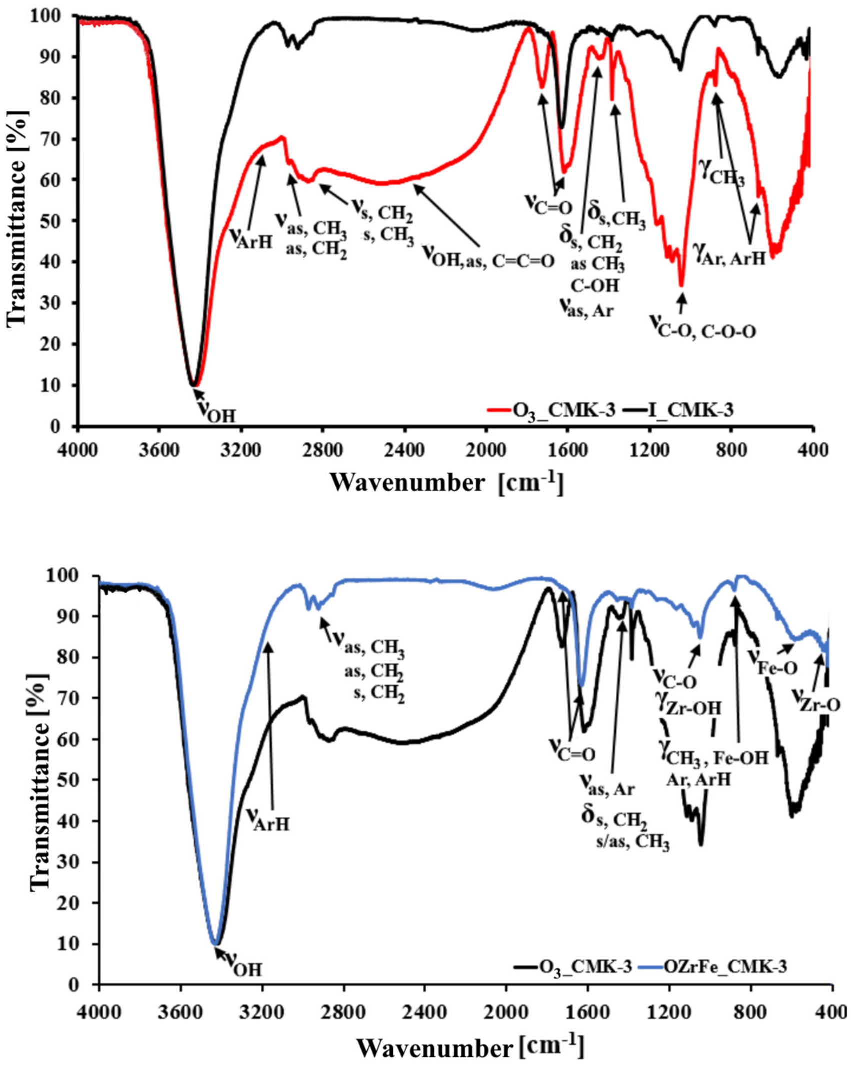

Figure S1. The FT-IR spectra of the I_CMK-3, O_3_CMK-3 and OZrFe_CMK-3.

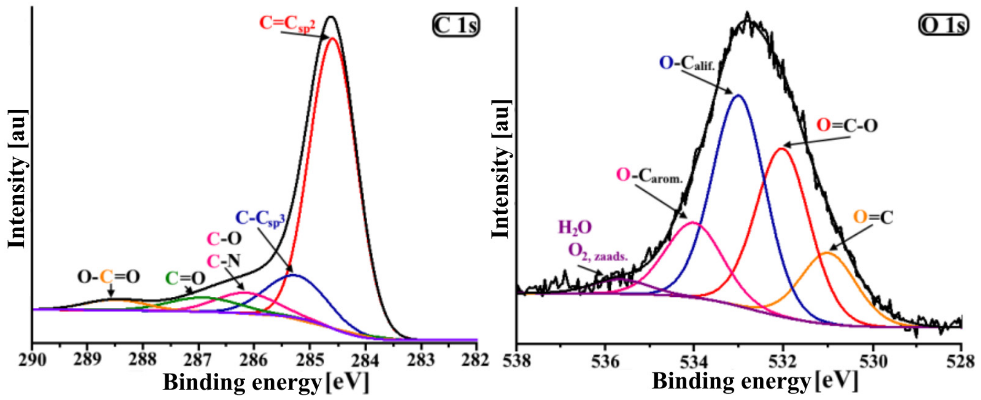

Figure S2. The high-resolution XPS spectra of the core energy levels C 1s and O 1s for O_3_CMK-3 carbon.

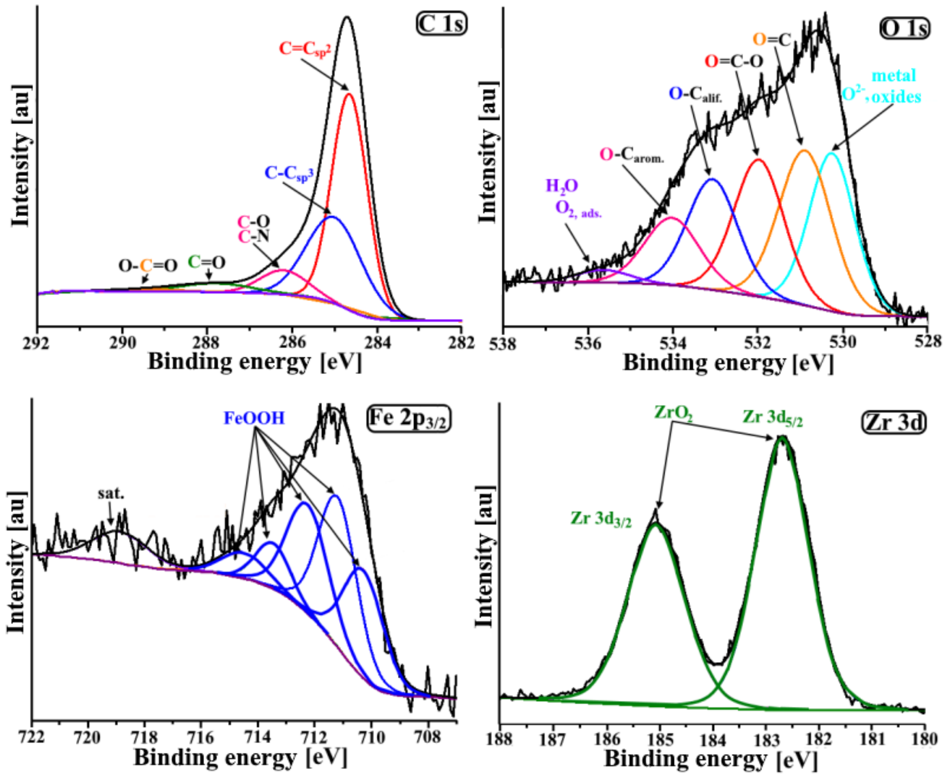

Figure S3. The high-resolution XPS spectra of the core energy levels C 1s, O 1s, Fe 2p3/2 and Zr 3d for OZrFe_CMK-3 carbon.

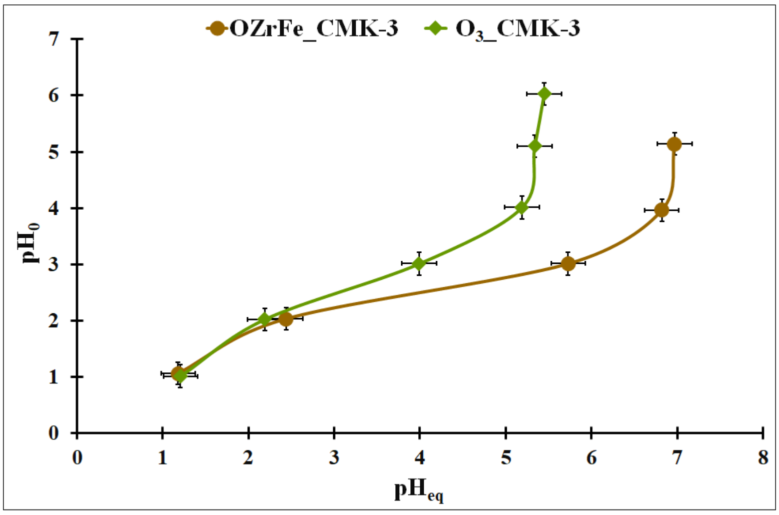

Figure S4. The pH_0 in the function of pH_{eq} for studied carbonaceous materials during Cr(VI) adsorption (m = 20 mg, V = 5 mL,
t = 24 hrs, $C_{0Cr(VI)}$ = 58 mg L^{-1}).

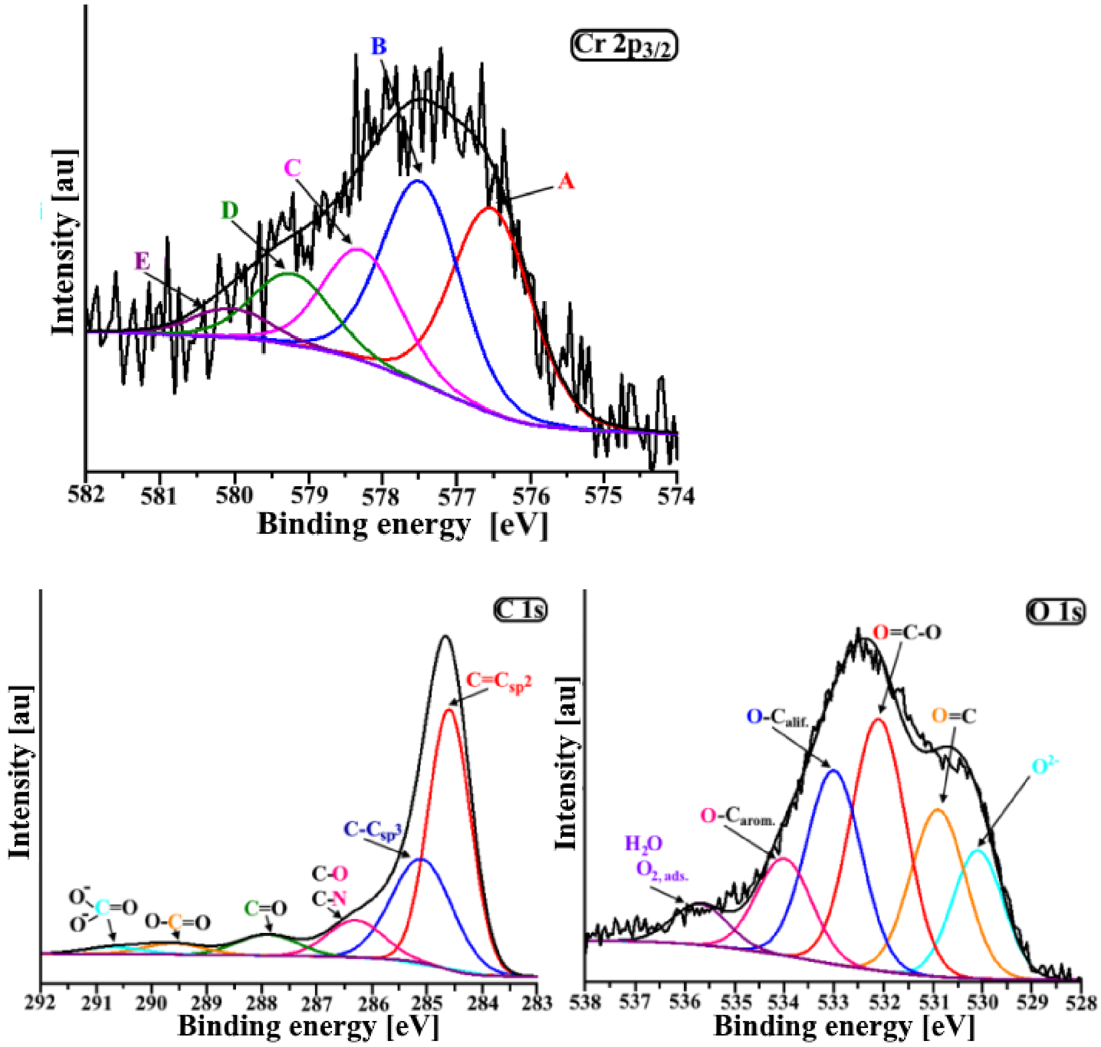

Figure S5. The Cr 2p3/2 energy core level for Cr-loaded OZrFe_CMK-3 carbon (the Cr content: 50 mg g^{-1}; A: 576.5 eV, B: 577.5 eV, C: 578.3 eV, D: 579.2 eV, E: 580.0 eV; this signal is related to the Cr(III)).

Table S1. The comparison of Cr(VI) adsorption performance between data obtained from the literature and our studies.

**Material**	**pH**	**a_{max} [mg g^{-1}]**	**Lit.**
Rice husk	5.0	38.4	[1]
Eshcherichia coli and *Staphylococcus epidermidis*	3.0-6.0	16.9	[2]
Fusarium sp. (filamentous)	5.0	18.2	[3]
O_3_CMK-3	5.3	29.9	our study
OZrFe_CMK-3	5.8	50.1	

[1] Gonzalez, M.H.; Araújo, G.C.; Pelizaro, C.B.; Menezes, E.A.; Lemos, S.G.; De Sousa, G.B.; Nogueira, A.R.A. Coconut coir as biosorbent for Cr (VI) removal from laboratory wastewater. J. Hazard. Mater. 2008, 159, 252–256.
[2] Sen, M.; Dastidar, M.G. Adsorption-desorption studies on Cr (VI) using non-living fungal biomass. Asian J. Chem. 2010, 22, 2331.
[3] Elangovan, R.; Philip, L.; Chandraraj, K. Biosorption of chromium species by aquatic weeds: Kinetics and mechanism studies. J. Hazard. Mater. 2008, 152, 100-112.
